# Turning a Substitute for Craniotomy

**Published:** 1847-09

**Authors:** 


*Turning a Substitute for Craniotomy.*—Dr. Simpson states that he has practised turning as an alternative for craniotomy and the long forceps, in several cases in which the head had been morbidly detained at the brim of the pelvis, from the slighter forms of disproportion between the two; and he believes it to present various advantages over embryulcio. It gives the child a chance of life; it is more safe to the mother, because it can be performed earlier in the labour, and more speedily; it enables us to adjust and extract the head of the child through the imperfect pelvic brim in the most advantageous form and direction, the head flattening *laterally* under the traction; the neck of the child (if it were living, or only lately dead,) is so strong as to allow us to exert such a degree of traction upon the obstructed head, that the sides of the cranium might become very greatly compressed, or even indented under it, and that without necessarily destroying the child; and, lastly, he observes, it is a practice which can be followed when proper instruments are not at hand, and the avoidance of instruments is generally desirable when it is possible.—*Prov. Med. and Surg. Journ. from Monthly Journ. of Med. Science.*

